# Molecular analysis of *Pseudomonas aeruginosa* isolated from clinical, environmental and cockroach sources by ERIC-PCR

**DOI:** 10.1186/s13104-018-3765-z

**Published:** 2018-09-15

**Authors:** Omid Zarei, Leili Shokoohizadeh, Hadi Hossainpour, Mohammad Yousef Alikhani

**Affiliations:** 1 Student Research Committee, Hamadan University of Medical Sciences, Hamadan, Iran; 2 Department of Microbiology, Faculty of Medicine, Hamadan University of Medical Sciences, P.O box: 6517838678, Hamadan, Iran

**Keywords:** *Pseudomonas aeruginosa*, ERIC-PCR, Clinical, Environmental, Cockroaches

## Abstract

**Objective:**

The objective of this study was to investigate the antibiotic susceptibility, virulence factors and clonal relationship among *Pseudomonas aeruginosa* isolated from environmental sources, hospitalized patients and the surfaces of cockroaches in the ICUs of four hospitals in Hamadan, west of Iran. A total of 237, 286 and 156 bacterial isolates were collected from clinical, environmental and cockroach sources respectively from May to September, 2017. The antimicrobial susceptibility was determined using disk diffusion method. The virulence factors, exotoxins A, S and U were detected by PCR. The genetic linkage of *P. aeruginosa* isolates were analyzed by Enterobacterial Repetitive Intergenic Consensus (ERIC)-PCR.

**Results:**

According to our findings, 58 (24.4%), 46 (16%) and 5 (3.25) *P. aeruginosa* were isolated from clinical, environmental and cockroach samples respectively. The MDR phenotypes were detected in 18 (45%) and 15 (37.5%) of clinical and environmental strains. The environmental isolates harbored more *exo*A and *exo*S than did clinical isolates. Genetic diversity was established among *P. aeruginosa* isolates as 14 different ERIC fingerprints were detected. The clonal relationships was detected among clinical, environmental and cockroach isolates. Our results highlighted the importance of identifying and controlling the potential sources of *P. aeruginosa* infections in hospitals.

## Introduction

*Pseudomonas aeruginosa,* an aerobic and positive oxidative gram-negative bacterium, is known as one of the most important causes of nosocomial infections especially in the intensive care units (ICU) [1]. This bacterium is not normally pathogenic but creates opportunistic infections in people with a weak immune system such as ICU patients [2, 3]. It is a ubiquitous organism, especially in environments such as soil, stagnant water, sediment, food, and in hospital settings [4]. Pathogenesis of *P. aeruginosa* is due to the production of several cellular virulence and extracellular factors. The most important extracellular factors of *P. aeruginosa* include exotoxin S (*exoS*), exotoxin U (*exoU*), exoenzyme A (*exoA*), secretion proteins III, elastase, alkaline protease, and IV protease, each of which has a toxic effect on mammalian cells [5, 6]. This bacterium accounts for around 13.5% of the infections of the Gram-negative bacteria in the ICU and is a primary cause of pneumonia associated with the ventilator in the ICU. The release of these bacteria in the ICU is difficult to control as they are resistant to many antibiotics by several mechanisms [7, 8].

There are numerous reports of *P. aeruginosa* disease outbreak that is attributed to environmental sources [9, 10]. Insects are known as the most common sources in the transmission, spread, and the number of diseases. Cockroaches can be infected with about 40 different species of vertebrate pathogens under natural or in vitro conditions [11–13]. The bacterial agents are located in different parts of the internal and external body of the cockroach and can survive for days making these insects a proper carrier and distributor of these agents to human [14]. For the purpose of epidemiological studies and to study genetic linkages of bacteria, especially in nosocomial infections, common methods of bacterial typing such as antibiotic resistance patterns, phage typing and serotyping have been replaced by molecular methods such as ribotyping, pulse field gel electrophoresis (PFGE) and PCR-based methods [15]. ERIC-PCR technique is one of the PCR-based methods in which the position and number of ERIC sequences which are different in bacteria are used as a genetic marker for bacterial diversity [15, 16].

The objective of this study is therefore to investigate the antibiotic susceptibility, virulence genes and genetic relationship among *P. aeruginosa* strains isolated from clinical, environmental and cockroach sources using ERIC-PCR technique.

## Main text

### Methods

#### Identification of *Pseudomonas aeruginosa* isolates

In a cross-sectional study, a total of 237 clinical samples of hospitalized patients in ICUs, 286 environmental samples of ICUs and 156 samples from external body of cockroaches were isolated from the four teaching hospitals in Hamadan, from May to September, 2017.

*Pseudomonas aeruginosa* isolates were identified and confirmed by conventional microbiological and biochemical tests [17]. Cockroaches were captured manually using Matchbox. Bacteria were isolated from Cockroaches by placing them in a solution containing peptone water. In brief, 500 μl of the peptone solution was inoculated in nutrient agar and blood agar plates. The inoculated plates were then incubated aerobically at 35 °C for 72 h [18].

#### Antibiotic susceptibility testing

The following antibiotics disks (Mast Group Co, UK) were used: gentamicin (GM, 10 µg), ciprofloxacin (CIP, 5 µg), imipenem (IMI, 10 µg), meropenem (MEN, 10 µg), colistin (CO, 10 µg), and piperacillin (PIP, 10 µg). Antibiotic susceptibility was determined by disk diffusion method, according to the Clinical and Laboratory Standard Institute (CLSI 2017) guidelines.

#### DNA extraction and PCR

Genomic DNAs were extracted from *P. aeruginosa* isolates by a commercial DNA extraction kit (Qiagen, Hilden, Germany). Virulence genes including *exo*A, *exo*S, *and exo*U were detected using specific primers as described previously [19, 20].

#### ERIC-PCR

The total 85 isolates which comprised of 40 clinical isolates, 40 environmental isolates and 5 isolates from cockroaches were selected for molecular typing by ERIC-PCR. This technique was carried out in a thermocycler (Bio-Rad, Inc. USA) using the primer ERIC (F): 5ʹ-ATG TAA GCT CCT GGG GAT TCAC-3ʹ and ERIC (R): 5ʹ-AAG TAA GTG ACTGGG GTG AGC G3ʹ (Pishgam Biotech Co, Iran) according to the following protocol: initial denaturation (94 °C for 5 min) followed by 40 cycles of denaturation (91 °C for 1 min), annealing (25 °C for 2 min), extension (72 °C for 2 min), and a final cycle of extension at 72 °C for 5 min. The PCR products were loaded on a 2% agarose gel (Sigma-Aldrich) at 70 V for 1 h, and the banding patterns were visualized on an ultraviolet illumination.

#### ERIC-PCR results analysis

The ERIC patterns were analyzed by online data analysis service (inslico.ehu.es). ERIC profiles were compared using Dice method and clustered by UPGMA program.

### Results and discussion

In view of the importance of *P. aeruginosa* in hospital settings, the antibiotic resistance patterns, virulence factors including *exo*A, *exo*U, and *exo*S and also the genetic linkage of *P. aeruginosa* from clinical, environmental and cockroach samples were investigated. In the current study, 58 (24.4%) *P. aeruginosa* isolates from clinical samples, 46 isolates (16.3%) from the environment of hospitals and 5 (3.2%) isolates from cockroaches were identified.

The Clinical isolates of *P. aeruginosa* were isolated from clinical samples including urine (12; 20%), burn wound (9; 15%), sputum (6; 10%), blood (5; 8%), CSF (4; 6%), eye (3; 5%) and ear (1; 2%). The environmental isolates of *P. aeruginosa* were identified in tracheal tube (8; 17%) ventilator and toilet (6; 13%), floor (4; 9%) bed sheet, trash bin and health worker hand (3; 6%), bathtub (2; 4%), sink, dosing container and keyboard (1; 2%). Several other factors like the compliance of health care workers have been shown to influence the rate of *P. aeruginosa* hospital-acquired infections, especially in high-risk units like ICUs [21]. In this study, some isolates of *P. aeruginosa* were recovered from hands of physicians and staff of ICUs. These isolates had MDR phenotypes and harbored virulence factors.

The isolation of *P. aeruginosa* from the cockroaches has been reported [22]; *P. aeruginosa gets* multiplied in the gut of the cockroaches and is then excreted for up to 114 days [21]. We isolated *P. aeruginosa* on the surfaces of cockroaches. *P. aeruginosa* isolated from cockroaches showed low virulence and antibiotic resistance.

**Fig. 1:**
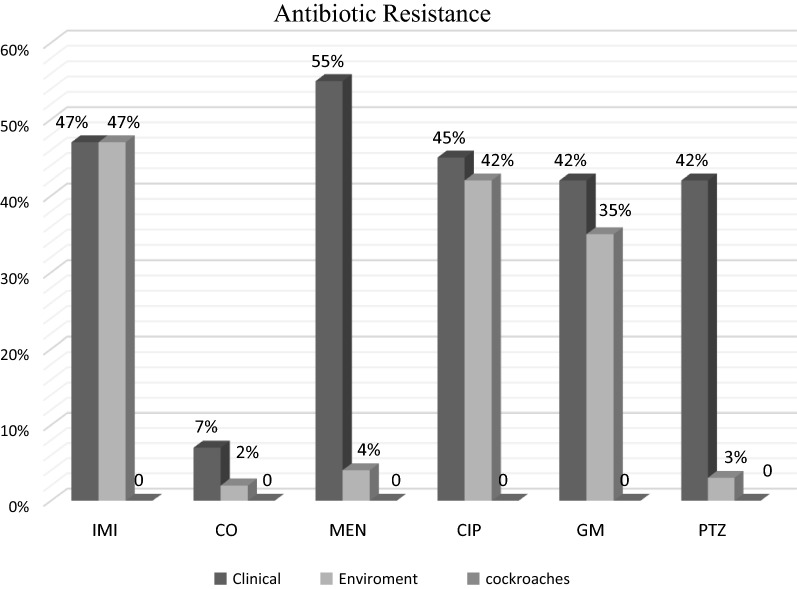
Antimicrobial resistance (%) of clinical, environmental and cockroaches *P. aeruginosa* isolates

The high-level resistant (55%) to meropenem was detected among clinical strains. Colistin was identified as the most effective antibiotics against *P. aeruginosa* from different sources. No resistance to antibiotics was detected in isolates from cockroaches. Clinical and environmental strains, however, revealed close susceptibility to antibiotics under study. The MDR phenotypes were detected in 18 (45%) and 15 (37.5%) of clinical and environmental strains. The results of antibiotic susceptibility testing is shown in Fig. 1.

*Pseudomonas aeruginosa* isolates showed high-level resistance to many antimicrobial agents. The high rates of clinical and environmental *P. aeruginosa* isolates show the multi-drug resistance (MDR) phenotype. Antibiotic susceptibility pattern of *P. aeruginosa* showed that 45% and 37.5% of clinical and environmental isolates were resistant to more than 3 antibiotics from different classes. However, if more antibiotics were checked, MDR isolates also would have been increased. According to our results, carbapenems (e.g., imipenem and meropenem), ciprofloxacin, gentamicin, and piperacillin did not have an effective activity against *P. aeruginosa* isolates. In line with our results, Mobaraki in Iran and Yi Dou in China reported the high-level resistance to ciprofloxacin and increase of multidrug-resistant strains of *P. aeruginosa* [23, 24]. Ding et al. [25] in a Meta-analyses of 50 studies published from 2010 to 2014 investigated antimicrobial-resistant *P. aeruginosa* and reported the varied prevalence of resistance, with high levels of resistance to gentamicin. Gonçalves et al. [26] reported that 73.9% of *P. aeruginosa* were multidrug-resistant and 43.9% were resistant to carbapenems. Khosravi et al. also found high-level resistance to gentamicin, ciprofloxacin, meropenem, piperacillin/tazobactam and imipenem, and no resistance to colistin among *P. aeruginosa* strains isolated from burn patients in a burn center of Ahvaz; Southeast of Iran [27].

We also found that there are some limitations in the use of ciprofloxacin, gentamicin and piperacillin, Imipenem and meropenem in *P. aeruginosa* infection therapy. Given the prevalence of resistant *P. aeruginosa* in hospital environments, medical equipment related to patients and hands of healthcare staff is probably serious causes of concern in hospitals. Where the use of β-lactam, aminoglycoside or quinolone is found to be quite ineffective, the polymyxins, especially colistin, would attract significant attention as antibiotics against MDR strains and as the final alternative treatment [28, 29]. Resistance to colistin is rarely observed and limited data are reported on the acquired resistance to colistin or other polymyxins. Since Colistin is thought to be the most effective antibiotic against MDR *P. aeruginosa*, the resistance of *P. aeruginosa* to colistin is increasing leading to serious challenges in the treatment of infections caused by MDR strains of this organism in hospitals, and clinical and environmental sections.

In this research, we studied the presence of three genes encoding virulence factors including *exo*A, *exoU*, and *exoS* in *P. aeruginosa.* The environmental strains harbored more frequent *exo*A and *exoS* genes than clinical strains. The frequency of *exo*U was higher in clinical strains. Only one strain from cockroaches contained *exo*A gene. The *exo*A+/*exo*S+ were the predominant (47.5%) genotype in environmental isolates. The frequency of virulence patterns among *P. aeruginosa* isolates from different sources are compared in Table 1.

**Table 1:** Virulence factor frequency in clinical and environmental *P. aeruginosa* isolates

Virulence factor	*exoA* *No (%)*	*exoS* *No (%)*	*exoU* *No (%)*	*exoA/exoS/exoU* *No (%)*	*exoA/exoS* *No (%)*	*exoA/exoU* *No (%)*
Clinical isolates	23 (57.5)	13 (32.5)	11 (27.5)	2 (5)	5 (12.5)	6 (15)
Environmental isolates	29 (72.5)	29 (72.5)	6 (15)	1 (2.5)	19 (47.5)	1 (2.5)
Isolates from cockroaches	1 (20)	0	0	0	0	0

There are varied reports on the frequency of *exo*A, *exo*U and *exo*S genes in different studies. Yousefi et al. reported the frequency of *exo*A, *exo*U and *exoS* genes as 90.4%, 66.7% and 65.4 in clinical isolates of *P. aeruginosa* in the South of Iran. Amirmozafar et al. detected the *exo*A and *exoS* strains in 81% and 61% of clinical isolates of *P. aeruginosa*. According to results from our study and others *exo*A is a more frequent virulence factor [30–32]. However, it is predominant in environmental isolates and less frequent in clinical isolates. *exo*U was only found in breathing aid equipment like ventilator and tracheal tube. No *exoU* was detected in eye infections. There was no significant relationship between virulence genes and clinical specimen type. *P. aeruginosa* strains possess a highly conserved genome which encodes genes important for survival in numerous environments and allows them to cause a variety of human infections [33].

**Fig. 2:**
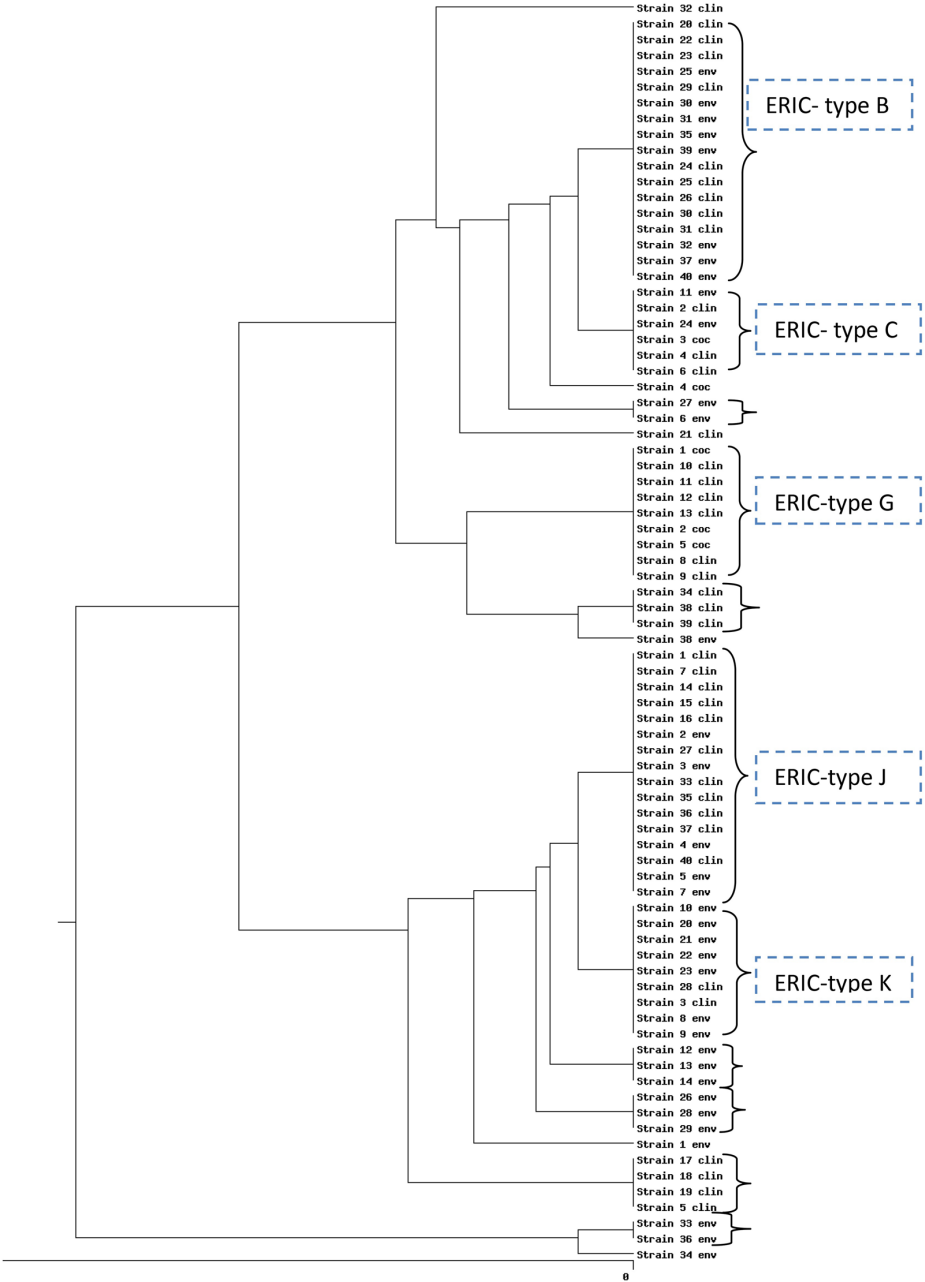
Dendrogram of ERIC-PCR analysis for 80 clinical and environmental *P. aeruginosa* isolates and 5 isolates from cockroaches in Hamadan hospitals. *E-Type* ERIC-Type, *clin* clinical, *env* environmental, *coc* cockroaches

Analysis of genetic linkage among isolates by ERIC-PCR showed 50–100% similarity among *P. aeruginosa* isolates (Fig. 2). Genetic diversity was established among *P. aeruginosa* isolates by detecting 14 different ERIC fingerprints with the similarity cutoff of ≥ 95%. 14 different ERIC profiles, including nine common types and five unique types, were identified. ERIC-type B as the predominant type comprised 17 isolates (42.3%) and E-type K comprised 16 isolates (40%). E-types C was shared by clinical isolates (urine and sputum), environmental isolates (tracheal tube and floor) and isolates from cockroaches. One clinical isolate from sputum and one isolate from the surface of cockroaches showed the same antibiotic resistance and virulence genes patterns. E-type K and J comprised clinical (burn wound, blood, CSF, urine, and sputum) and environmental (the tracheal tube, physician’s hand, sink, and ventilator) isolates. ERIC-type G was shared by clinical isolates (sputum, CSF, and blood) and isolates from cockroaches (Fig. 2). E-types C, K, and G were identified in the same hospital. This indicated the intra hospital dissemination of these clones. Our results also showed that the variation in the ERIC regions is not closely related to the presence of resistance or virulence genes and there was no significant relationship among antibiotic resistance, virulence gene and ERIC Patterns. We also found that isolates with common ERIC types showed different antibiotic resistance and virulence factor patterns. These findings indicate the presence of various isolates in terms of antibiotic resistance and virulence in hospitals.

Several studies have thus reported genetic diversity and heterogeneity among *P. aeruginosa* isolates using ERIC-PCR, rep-PCR, RAPD-PCR, PFGE, MLST, DLST methods in hospitals of Iran and other countries [10, 34, 35]. In this study, ERIC-PCR was used since it is much cheaper and easier to perform and adequate reliability, rapidity and discriminatory power have been documented for the typing of *P. aeruginosa* strains through this method [36].

In conclusion, ERIC-PCR analysis showed that there is clonal relatedness among clinical and environmental isolates and isolates taken from cockroaches in the ICUs. Diversity was also found among *P. aeruginosa* isolates in hospitals of Hamadan, Iran. Better understanding of the role of reservoirs in *Pseudomonas* infections by molecular typing methods would result inn better plans to minimize the transmission of the bacterial infections from patients to the environment and vice versa.

## Limitations

One of the most important limitations of this study was the low number of *P. aeruginosa* isolated from cockroaches. More sampling is required for molecular studies. We have also limitations in financial support.

## Abbreviations

*P. aeruginosa*: *Pseudomonas aeruginosa*
MEM: meropenem
IMI: imipenem
CIP: ciprofloxacin
GM: gentamicin
PTZ: piperacillin/tazobactam
CO: colistin
PCR: polymerase chain reaction
*exoA*: exotoxin A
*exoS*: exoenzyme S
*exoU*: exoenzyme U
